# Apoptotic Processes Precede Infection with Symbionts in a Pogonophoran Lavrae (Siboglinidae, Annelida)

**DOI:** 10.1134/S0012496622050118

**Published:** 2022-10-27

**Authors:** N. N. Rimskaya-Korsakova, E. N. Temereva, V. V. Malakhov

**Affiliations:** Moscow State University, Moscow, Russia

**Keywords:** frenulate pogonophorans, symbiotic bacteria, infection, Siboglinidae, Frenulata

## Abstract

The fine structure of the body wall and gut was for the first time studied in the competent larvae of the frenulate pogonophoran *Siboglinum fiordicum*. Mass apoptosis of cell nuclei was observed in the dermo-muscular body wall and coelomic epithelium. Apoptotic nuclei were found in both cell cytoplasm and outside of the larval body. In the latter case, each nucleus was surrounded by the plasmalemma, and the entire cluster was covered with the cuticle. Cells of the larval gut retained the usual structure with the cytoplasm filled with numerous yolky granules and the nucleus displaying usual morphology. Similar apoptotic processes have been described in vestimentiferans and found to be initiated by penetration of symbiotic bacteria through the integument into the dorsal mesentery. The process of apoptotic rearrangement of body wall cells and the formation of unique symbiosis with bacteria were assumed to be time-spaced in *S. fiordicum*, occurring sequentially rather than simultaneously, unlike in vestimentiferans.

Pogonophorans, or frenulate pogonophorans (Annelida: Siboglinidae), are tubeworms and inhabit reduced environments, including methane seeps, the periphery of hydrothermal vents, organic-rich sediments, etc. In the mid-20th century, frenulate pogonophorans were considered as a separate animal phylum that is closely related to deuterostomes [1]. To date, morphological, molecular, and phylogenetic studies have reliably established the position of pogonophorans within the phylum Annelida among other Siboglinidae, which include the well-known groups Vestimentifera and *Osedax* bone-eating worms [2–4]. Frenulate pogonophorans (Frenulata) are a sister clade to all other Siboglinidae, thus being a crucial group for studying the structure and development of Siboglinidae.

The most unusual feature of Siboglinidae is that adults lack a mouth and a gut and derive their nutrition from symbiotic bacteria, which live in trophosome cells [5]. Bacteria are not vertically transmitted from the mother to developing oocytes, but are acquired horizontally from the environment by settled larvae [6]. Infection with bacteria occurs through the tegument of the trunk portion of the larval body in vestimentiferans, which arose more recently than pogonophorans in evolution. Bacteria facilitate proliferation of mesodermal cells, which form the trophosome, an organ of symbiotrophic nutrition. The gut, which functioned earlier, is degraded, and the skin and other tissues through which bacteria have entered the body die via cell apoptosis [7]. In the case of frenulate pogonophorans, it is still believed that bacteria enter the larval body through the mouth and colonize the gut epithelium, which is then transformed into the trophosome [8].

Owing to their unique symbiosis with bacteria, Siboglinidae worms colonize habitats with extreme conditions, such as hydrothermal vent craters (vestimeniferans), whale bones (*Osedax*), and hydrocarbon seeps (vestimeniferans and pogonophorans). The establishment of symbiosis is still an enigma, and new data are necessary to obtain for understanding the evolution of the group and the origin of this unique phenomenon.

 The objective of this work was to study the ultrastructures of the dermo-muscular body wall, coelomic epithelium, and gut in settling larvae of the frenulate pogonophoran *Siboglinum fiordicum* Webb, 1963, in order to understand the state in which larval tissues occur prior to being infected with symbiotic bacteria.

To study the ultrastructure of competent *S. fiordicum* larvae, experiments with larval settlement were carried out in laboratory conditions. Marine mud and maternal *S. fiordicum* worms were collected with a grab sampler from a depth of 35 m near the Espergrend Marine Biological Station, University of Bergen, Norway, in September 2018. Larvae isolated from maternal tubes varied in age from trochophores to competent larvae and in size from 200 to 500 µm. The larvae were transferred into dishes with mud collected together with maternal worms and were kept at 6°C [9, 10]. Competent larvae with a body length of 800 µm were observed after several weeks of culturing. Three competent larvae were fixed with 3% glutaraldehyde in sea water, postfixed with 1% osmium tetroxide in sea water, and embedded in Spurr’s resin. Thin sections were obtained using a Leica EM UC7 microtome (Leica Microsystems, Wetzlar, Germany) with a DIATOM diamond knife (Diatome Ltd., USA). The thin sections were transferred onto copper blends and contrasted with uranyl acetate and lead citrate. The preparations were examined with a JEOL JEM 1011 transmission electron microscope (JEOL, Japan).

The body length is approximately 800 µm in premetamorphose larvae (Fig. 1a). The body consists of a prostomium, a peristomium, a forepart (the second segment with a cuticular frenulum and a tentacular anlage), a trunk (the third segment with two rows of annular chaetae), more than six opisthosomal segments, and a pygidium (Fig. 1a). The larva has the ciliary bands prototroch, neurotroch (ventral ciliary band), and mesotroch and chaetae on the fist opisthosomal segment and annula (Fig. 1a). The larval body is coated with a cuticle.

**Fig. 1.**
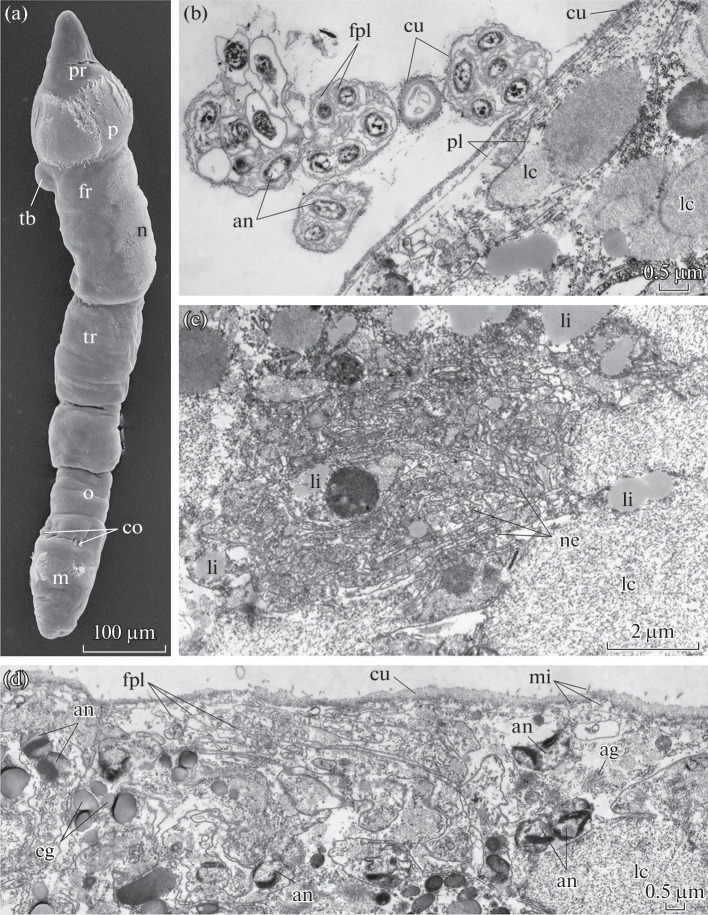
(a) Overview and (b–d) fine structure of the integument in *S. fiordicum* larvae before metamorphosis. (a) Appearance of a larva, scanning electron microscopy. (b) Clusters of one to seven apoptotic nuclei ejected from the body wall. Each nucleus is surrounded with a shrunken (folded) plasmalemma, and the total cluster is surrounded with a cuticle. Transmission electron microscopy. (c) Normally developed intraepidermal circumesophageal connectives, transmission electron microscopy. (d) Swollen cells of the epidermis of the body wall contain loose material; cell membranes form numerous folds; fragmented or whole apoptotic nuclei occur at the cell periphery. Transmission electron microscopy. Designations: ag, Golgi apparatus; an, apoptotic nuclei; ca, chaetae of the annula; co, chaetae of the first segment of the opisthosoma; cu, cuticle; eg, electron-dense granules; f, cuticular frenulum; fr, forepart segment; fpl, shrunken plasmalemma; lc, loose (reticulate or fibrous) cytoplasm; li, lipid drops; m, mesotroch; mi, microvilli; n. nerotroch (ventral ciliary band); ne, neurites; o, first segment of the opisthosoma; p, prototroch; pe, peristomium; pl, plasmallema; pr, prostomium; py, pygidium; tb, tentacle rudiment; tr, trunk segment.

A study of the fine structure of the epidermis showed that the cell shape was substantially altered. Cells appeared to be swollen and filled with loose (reticulate or fibrous) material. The epithelial polarity was distorted; i.e., the basement membrane was detectable only in some regions of the epithelium and cell contacts were lacking (Figs. 1b–1d). Cell membranes formed numerous folds (Figs. 1b, 1d). Fragmentary or whole apoptotic nuclei were observed at the cell periphery and had apoptosis-specific fine structures; i.e., the nuclear membrane was often distorted and chromatin formed electron-dense peripheral bands (Fig. 1d). Unusual bodies were found over the larval body surface, representing clusters of nuclei ejected from the larval body wall (Fig. 1b). Each body is covered with the cuticle and contains clusters of one to seven fragmented nuclei, which are each surrounded with the shrunken plasma membrane (Fig. 1b).

Longitudinal and circular body muscles, which are characteristic of larvae of younger stage, were absent at this stage, as well as specialized muscles of chaetae and the first septum. Presumable muscle cells were degraded and lacked nuclei and myofilaments; their cytoplasm was filled with fibrous loose material. Coelomic epithelial cells also underwent degradation. Multicellular tubiparous glands were preserved at this stage, and their ducts, which were lined with a microvillar epithelium, could be tracked between degenerating cells of the body wall. The lumens of the tubiparous glands appeared filled with electron-lucent secretion of the prospective tube. Normally developed intraepidermal nerve cords were detectable in the integument of the prostomium (Fig. 1c). The larva had a gut, a miniature mouth funnel, and a terminal anal fossa. There was no lumen in the gut. Gut cells contained yolky material to such an extent that cell surfaces were close together within the inner gut lumen, and the lumen was consequently undetectable.

 Bacteria have been shown to infect larvae through the integument of the trunk segment in a recent study of vestimentierans by transmission electron microscopy and molecular-genetic fluorescence in situ hybridization (FISH) [7]. Bacteria penetrate through the skin, enter the dorsal mesentery between the dorsal blood vessel and the gut, and stimulate proliferation of dorsal mesenterial cells. These numerous cells are colonized by bacteria and form a trophosoma. Apoptosis is observed in the epidermis and all internal tissues that bacteria utilize to enter the body [7]. Thus, colonization by bacteria and tissue renewal via apoptosis occur simultaneously in vestimentiferans. The gut is degraded almost completely and ceases functioning, and the trophosome forms de novo from tissues of a mesodermal origin.

Our findings demonstrate that skin, muscle, and coelomic epithelial tissues undergo apoptotic processes in settling larvae of the frenulate pogonophoran *S. fiordicum* before metamorphosis. Some nuclei are fragmented and lysed within epidermal cells, while some apoptotic nuclei are ejected from the body. The cell cytoplasm is filled with loose matter, and the majority of cell organelles are destroyed. Bacteria are not found in the skin at this stage. We assume that apoptotic processes in the epidermis and mesodermal tissues precede tissue colonization with potential symbiotic bacteria in frenulate pogonophorans.

Endodermal tissues retain their larval state in the process. Cells of the larval gut are filled with yolky material and remain unchanged. The mouse and anus are present, but are not connected with the gut. The feature indicates that the gut is not utilized in larvae to assimilate new substances from the environment through the mouth, but serve as a depot to store the yolk originating from the maternal body. The anatomy of the digestive tract, the integrity of the gut epithelium, and the lack of symbiotic bacteria in the gut indicate that an alimentary mechanism does not mediate bacterial colonization and that bacteria enter the body through the integument, like in vestimentiferan larvae.

To summarize, degradation of the skin and muscles precedes bacterial colonization of the larval body in frenulate pogonophorans, which are a sister group to other Siboglinidae. A comparison of the infection process between pogonophorans and vestimentiferans shows that bacterial penetration, a renewal of tissues through which symbiotic bacteria enter the body, and the start of trophosoma formation occur simultaneously in vestimentiferans, which are evolutionarily younger than pogonophorans.
